# Predictors of Severity of Alcohol Withdrawal in Hospitalized Patients

**DOI:** 10.4021/jocmr1514w

**Published:** 2013-08-05

**Authors:** Radhames Ramos, Thierry Mallet, Anthony DiVittis, Ronny Cohen

**Affiliations:** a Nassau County Medical Center, East Meadow, New York, USA; b Department of Medicine - Woodhull Medical Center, Brooklyn, New York, USA; c Statistics - Woodhull Medical Center, Brooklyn, New York, USA; d NYU School of Medicine. New York, New York, USA.; 7

**Keywords:** Alcohol withdrawal, Delirium tremens, Seizures, Detoxification

## Abstract

**Background:**

Alcohol withdrawal is a relatively common problem among chronic alcohol users, and its severity will determine the setting in which it will be more appropriate to take care of the patients. Those with mild symptoms will be managed in an outpatient setting, as opposed to those with advanced moderate or severe symptoms who will require inpatient management. Among those patients who will require hospitalization, some of them will do well in a regular floor, but some of them will have to be managed in an intensive care unit. We tried to determine whether some variables could be predictive of an increased risk of being managed in an intensive care unit as opposed to being managed in a regular medical floor.

**Methods:**

A retrospective non-randomized review trial design was implemented and a total of 110 medical charts of patients admitted to our institution with severe alcohol withdrawal during the calendar year of 2009 were reviewed. Different demographic and clinical parameters were analyzed, and their significance established in regard to the clinical settings (ICU vs. medical floor) in which the patients were managed.

**Results:**

The patients managed in the ICU were found to be younger than their counterparts who were managed in the medical floor, and they were more likely to be white and unemployed. On the other hand, being diabetic, using over-the-counter drugs or prescribed medications appeared to be protective factors, resulting in management of alcohol withdrawal on the medical floors.

**Conclusion:**

A likely explanation to our findings could be that patients exhibiting better health protective behaviors have a better chance to stay away from the ICU. However no tools could be developed to stratify the patients’ risks and more behavioral and observational cohort studies will be needed for that purpose.

## Introduction

Unhealthy Alcohol Use and Alcoholism are such common problems that virtually every clinician will eventually have to deal with their complications. Complications are likely to happen when an otherwise “healthy” alcoholic suddenly stops its consumption, which may result in the alcohol withdrawal syndrome that can present as mild, moderate or severe, including the most severe form, “Delirium tremens” (DT) [1, 2].

The alcohol withdrawal syndrome can develop in chronic alcoholics who are alcohol-dependent. Alcohol dependence includes physical dependence, tolerance and compulsive alcohol use that become the main priority of the subject. Signs and symptoms typically develop in alcohol-dependent people 6 - 24 hours after their last drink and may occur unintentionally if abstinence is enforced by illness, injury or other causes. The most common features are tremulousness, sweating, nausea, vomiting, anxiety and agitation. Delirium tremens, which is characterized by auditory and visual hallucinations, confusion, disorientation, clouding of consciousness, impaired attention, pronounced autonomic hyperactivity, develops. Neuronal excitation, including epileptiform seizures, can also occur. If left untreated, death by respiratory and cardiovascular collapse may result [1, 3].

Certain patients should be considered for inpatient treatment regardless of the severity of their symptoms. Relative indications for inpatient alcohol detoxification are history of severe withdrawal symptoms, history of withdrawal seizures or delirium tremens, multiple previous detoxifications, concomitant psychiatric or medical illness, recent high levels of alcohol consumption, pregnancy, and lack of a reliable support network [4, 5]. The revised Clinical Institute Withdrawal Assessment for Alcohol (CIWA-Ar) scale is a validated 10-item assessment tool that can be used to quantify the severity of alcohol withdrawal syndrome [4] and enables the provider to classify the patient into three different categories: mild (not requiring hospitalization), moderate and severe. The most severe forms of withdrawal, impending DT, require intensive monitoring and a need to be followed in a high intensity care unit.

It would be clinically useful to identify what variables will be predictive of which patients are at higher risk of ending up in the ICU due to severe alcohol withdrawal as opposed to being managed on the regular medical floor.

### Objective of the study

To determine which patients admitted to our institution for alcohol withdrawal will be at greater risk to develop severe alcohol withdrawal that will necessitate admission to the ICU. The goal is to identify the variables that would predict those patients with alcohol-related illness who would be more likely to need care in the ICU. This will allow us to monitor those patients more closely when admitted to the medical service.

## Methods and Materials

A retrospective non-randomized review trial design was implemented. The sample comprised every patient admitted to the Medical service diagnosed with severe alcohol withdrawal in our institution in calendar year 2009 (January 1, 2009 through December 31, 2009). A data extraction tool was designed, allowing reviewers to assess each chart for the following parameters: 1), Co-morbidities (dichotomous variable: present vs. absent): a), History of Diabetes; b), History of Hypertension; c), History of Dyslipidemia; d), History of Liver Disease; e), History of Renal Disease; f), History of Thyroid Disorder; g), History of Renal Disease; h), Psychiatric History; i), History of Allergies. 2), Laboratory Test Results: a), White blood cell count; b), Hemoglobin count; c), Hematocrit count; d), Platelet count; e), Glucose level; f), Blood alcohol level. 3), Demographic variables: a), Sex; b), Race/Ethnicity; c), Age. 4), Drug use history (dichotomous variables: yes/no): a), Over the counter; b), Prescription medication; c), Illegal drug use. 5), Current drug use frequency: a), Number of over the counter medications used; b), Number of prescription medications used; c), Number of illegal drugs used.

### Analysis plan

All data was extracted from the charts and placed in an Excel spreadsheet. Data was analyzed using SPSS (Statistical Package for the Social Sciences) software. Analyses included: 1), Frequency analysis; 2), Chi Square; 3), Regression; 4), Correlation.

## Results

A total of 110 medical charts of patients admitted to the medical service at Woodhull Medical and Mental Health Center with a diagnosis of Alcohol withdrawal between January 1 and December 31, 2009 were reviewed. Fifty-nine of the patients had to be managed in the ICU (53.6%), whereas 51 patients were managed on the regular medical floor during their entire stay. The younger patients were most likely to have been managed in the ICU (13 out of 16 patients (81.25%) below age 40 were managed in the ICU. Seventy percent of diabetic patients were managed on the medical floor and the remaining 30% in the ICU. The vast majority (93.2%) of those who were unemployed were managed in the ICU, whereas 91.7% of the patients who were employed were managed on the medical floor. Among those managed on the medical floor, 70.6% of the patients were taking prescription drugs, as opposed to only 49.2% of those managed in the ICU. Nearly two thirds (64.7%) of the patients managed on the medical floor were taking OTC drugs, as opposed to only 15.3% of those managed in the ICU. The distribution of sex and most of the comorbidities, excluding diabetes, was similar between the 2 groups. See Table 1 for a demographic description of the sample.

**Table 1:** Demographic Description of the Sample

	ICU		General Floor	

Variable	n	percent	n	percent
Age				
20 - 29	2	3.40%	0	0%
30 - 39	11	18.6	3	5.9
40 - 49	14	23.7	15	29.4
50 - 59	25	42.4	19	37.3
60 - 69	5	8.5	5	9.8
70 +	2	3.4	9	17.6
Totals	59	100	51	100
Sex				
Male	54	91.5%	44	86.30%
Female	5	8.5	7	13.7
Totals	59	100	51	100
Race/Ethnicity				
Hispanic	17	29.80%	28	66.70%
Black/African American	8	14	6	14.3
White	31	54.4	8	19
Asian Indian	1	1.8	0	0
Totals	57	100	42	100
Employment				
Unemployed	55	94.83%	4	10.81%
Employed	3	5.17	33	89.19
Totals	58	100.00	37	100
Prescription Drug Use				
Yes	29	49.20%	36	70.60%
Over the Counter Drug Use				
Yes	9	15.30%	33	64.70%
Illegal Drug Use				
Yes	4	6.78%	7	13.73%
Comorbidities				
History of hypertension	27	458%	18	35.30%
History of diabetes	6	10.2	14	27.5
History of dyslipidemia	5	8.5	5	9.8
History of liver disease	7	11.9	8	15.7
History of renal disease	3	5.1	1	2
Psychiatric history	10	16.9	11	21.6
History of thyroid disorder	1	1.7	1	2
History of allergies	2	3.4	2	3.9

The average age of the participants was 51.25 years, (sd = 11), with patients in the ICU being significantly younger than their counterparts on the general floor (48.42 years vs. 54.53 years respectively, t -3.006, P = 0.003).There were no differences between the groups in terms of Body Mass Index (BMI), white blood cell count, hemoglobin, hematocrit, platelet count, glucose level and blood alcohol level (Table 2).

**Table 2:** Differences Between Group Means

Variable	ICU		General Floor		Analysis	

	Mean	SD	Mean	SD	t	P
Age	48.42	10.39	54.53	10.89	-3.006	0.003
Body Mass Index	26.25	4.96	26.58	6.06	-0.294	NS
White blood cell count	6.45	2.91	6.67	2.75	-0.403	NS
Hemoglobin count	13.51	1.99	13.26	2.08	0.647	NS
Hematacrit count	38.78	5.54	38.40	6.19	0.647	NS
Platelet count	171.15	99.73	193.18	93.95	0.333	NS
Glucose level	107.05	27.04	119.78	48.97	-1.707	NS
Blood alcohol level	178.44	185.51	179.73	153.35	-0.39	NS

We examined the relationship between level of care (ICU vs. General Floor) and the patient’s health history and co-morbidities (Table 3). Details of their significance follow below in the discussion section.

**Table 3:** Relationship Between History/Comorbidities and Assignment to the ICU

Variable correlated with ICU	Chi Square	P
Employment	67.75	< 0.0005
Hypertension	1.24	NS
Sex	0.776	NS
Race/Ethnicity	14.84	0.001
Diabetes Mellitus	5.49	0.019
History of Dyslipidemia	0.06	NS
History of Liver Disease	0.34	NS
History of Renal Disease	0.76	NS
History of Obesity	0.38	NS
Psychiatric History	0.48	NS
History of Allergies	0.22	NS
Use of Illegal Drugs	1.47	NS
Use of Over the Counter Drugs	28.34	< 0.0005
Use of Prescription Drugs	5.2	0.023

## Discussion

Analysis of the data revealed that there was a statistically significant difference for 6 variables: age, employment status, race/ethnicity, diabetes mellitus, use of over the counter medications, and use of prescription drugs. Patients in the ICU were significantly younger than their counterparts on the general floor (48.42 years vs 54.53 years, P = 0.003). There was a strong correlation between unemployment and being managed in the ICU (P < 0.0005). There was also a strong correlation between race/ethnicity and severe alcohol withdrawal requiring ICU management, with Caucasian patients having to be managed in the ICU significantly more than anyone else (P = 0.001). On the other hand, there was a strong correlation between having Diabetes mellitus and being managed on the medical floor (P = 0.019). The same finding holds for use of over the counter drugs (P < 0.0005) and prescription drugs (P = 0.023) and being managed on the medical floor. Otherwise the 2 groups were similar in terms of sex, body mass index, basic laboratory values and all the other comorbidities encountered. There was no statistically significant difference in the other variables analyzed in the study.

What can we take from the analysis of this data? The fact that white patients are significantly more likely to require ICU admission might be a marker variable for ethnicity. Our community represents a large, immigrant Polish population seeking care in our hospital. This could be a reflection of a high rate of alcohol consumption and alcoholism among young Polish immigrants. This coupled with high rates of unemployment and sometimes homelessness, we have a socio-economic background that is ripe for the development of severe alcohol withdrawal. On the other hand, patients exhibiting health protective behaviors as a result of a history of chronic illnesses, particularly diabetes, who have to take medications in order to stay healthy, seem to be somewhat protected from the deleterious consequences of alcohol withdrawal.

### Conclusion

Alcohol withdrawal is a very dynamic entity that evolves in a matter of hours. It can range from a mild to a very severe, including “Delirium Tremens”, entity that if goes untreated might carry important mortality rates. In our institution, several of the patients (53.6%) diagnosed with alcohol withdrawal who were initially admitted to the medical floor had to be transferred to the Intensive Care Unit (ICU) where they would be managed more aggressively. Our study tried to determine if those patients followed a typical pattern.

Predictors indicating a higher likelihood of developing a more severe withdrawal, and hence increasing one’s chance of being treated in the ICU were: white race, younger age and unemployment. Conversely being diabetic, using over the counter drugs or prescribed medications appeared to be protective factors.

These findings cannot be fully explained, and considering the fact that this was not a large scale study, it would be extremely difficult to develop a scoring tool from it. A likely explanation would be that patients exhibiting better health protective behaviors are less likely to become very sick from alcohol withdrawal and less likely to necessitate management in the ICU, whereas young patients from the white immigrant Polish population, tend to become unhealthy alcohol users, unemployed and at higher risk to develop more severe withdrawal likely necessitating care in the ICU. This certainly provides opportunities to pursue more behavioral, observational cohort studies that could further explain our findings and confirm if such a trend would exist. This should be done as a prospective, open-trial instead of a retrospective one. Hopefully this study will enlighten us in knowing better how to recognize which patients are at higher risk of developing very severe alcohol withdrawal.
